# Exploring the influence of focused ion beam processing and scanning electron microscopy imaging on solid-state electrolytes

**DOI:** 10.1093/jmicro/dfac064

**Published:** 2022-11-21

**Authors:** Ziming Ding, Yushu Tang, Venkata Sai Kiran Chakravadhanula, Qianli Ma, Frank Tietz, Yuting Dai, Torsten Scherer, Christian Kübel

**Affiliations:** Institute of Nanotechnology (INT), Karlsruhe Institute of Technology (KIT), Eggenstein-Leopoldshafen 76344, Germany; Institute of Materials Science, Technische Universität Darmstadt, Darmstadt 64289, Germany; Institute of Nanotechnology (INT), Karlsruhe Institute of Technology (KIT), Eggenstein-Leopoldshafen 76344, Germany; Skyroot Aerospace Pvt. Ltd., Hyderabad, Telangana, India; Institute of Energy and Climate Research, Materials Synthesis and Processing (IEK-1), Forschungszentrum Jülich GmbH, Jülich 52425, Germany; Institute of Energy and Climate Research, Materials Synthesis and Processing (IEK-1), Forschungszentrum Jülich GmbH, Jülich 52425, Germany; Institute of Nanotechnology (INT), Karlsruhe Institute of Technology (KIT), Eggenstein-Leopoldshafen 76344, Germany; Institute of Materials Science, Technische Universität Darmstadt, Darmstadt 64289, Germany; Institute of Nanotechnology (INT), Karlsruhe Institute of Technology (KIT), Eggenstein-Leopoldshafen 76344, Germany; Institute of Nanotechnology (INT), Karlsruhe Institute of Technology (KIT), Eggenstein-Leopoldshafen 76344, Germany; Institute of Materials Science, Technische Universität Darmstadt, Darmstadt 64289, Germany; Helmholtz Institut Ulm (HIU), Karlsruhe Institute of Technology (KIT), Eggenstein-Leopoldshafen 76344, Germany; Karlsruhe Nano Micro Facility (KNMF), Karlsruhe Institute of Technology (KIT), Eggenstein-Leopoldshafen 76344, Germany

**Keywords:** focused ion beam, scanning electron microscopy, beam damage, Au coating, cryogenic condition, solid-state electrolyte

## Abstract

Performing reliable preparation of transmission electron microscopy (TEM) samples is the necessary basis for a meaningful investigation by *ex situ* and even more so by *in situ* TEM techniques, but it is challenging using materials that are sensitive to electron beam irradiation. Focused ion beam is currently the most commonly employed technique for a targeted preparation, but the structural modifications induced during focused ion beam preparation are not fully understood for a number of materials. Here, we have investigated the impact of both the electron and the Ga^+^ ion beam on insulating solid-state electrolytes (lithium phosphorus oxynitride, Na-β"-alumina solid electrolyte and Na_3.4_Si_2.4_Zr_2_P_0.6_O_12_ (NaSICON)) and observed significant lithium/sodium whisker growth induced by both the electron and ion beam already at fairly low dose, leading to a significant change in the chemical composition. The metal whisker growth is presumably mainly due to surface charging, which can be reduced by coating with a gold layer or preparation under cryogenic conditions as efficient approaches to stabilize the solid electrolyte for scanning electron microscopy imaging and TEM sample preparation. Details on the different preparation approaches, the acceleration voltage dependence and the induced chemical and morphological changes are reported.

## Graphical Abstract

**Figure:**
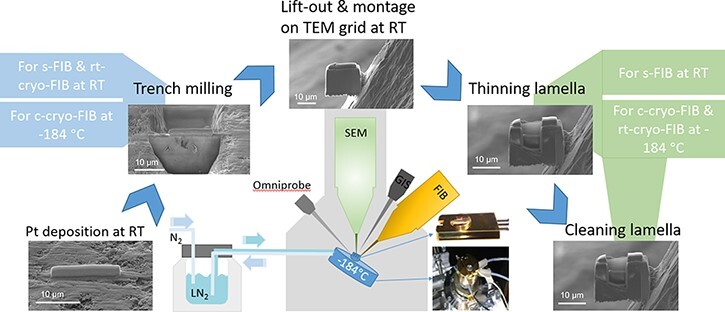

## Introduction

All-solid-state batteries (ASSBs) have a promising application potential due to their high safety, in part due to the non-flammable nature of solid-state electrolytes (SSEs) and their good mechanical stability, the potential for fast charging/discharging and the high-energy density with metallic anodes [1–3]. However, solid–solid interfaces, both in-between electrode and electrolyte and within, are still a major bottleneck for ASSBs to enter the market [4]. To understand and visualize the solid–solid interfaces, electron microscopy including scanning electron microscopy (SEM) and transmission electron microscopy (TEM) coupled with electron and X-ray spectroscopy is one of the most advanced characterization techniques to understand their morphology, structure, composition and oxidation state either during static imaging or dynamically at high resolution down to the atomic scale [5]. However, accessing the interfaces and producing electron-transparent specimens of the region of interest for TEM analysis without altering or damaging their structure is one of the obstacles to discover the story of materials degradation during battery cycling. A variety of approaches for SEM and TEM sample preparation of different electrode materials [6–8] and solid–solid interfaces [9,10] have been reported in the literature mainly including mechanical methods (e.g. using ultramicrotome [6] and diamond-tipped pen [10]), broad ion beam polishing (e.g. ion slicer [8,11]) and focused ion beam (FIB) [12–14] as well as the combination of FIB and ultramicrotomy [7]. Mechanical and broad ion beam polishing methods are preferred for the preparation of samples with large dimensions but can be challenging for brittle and air-sensitive materials, such as oxide solid electrolytes [9], or for a targeted preparation from a specific area. Alternatively, FIB-based TEM sample preparation techniques are currently probably the most employed approaches and have been used to look at a variety of SSEs such as LiPON- [15], oxide- [16] and sulfide-based [17] and lithium (Li) and fluoride [18] fast ion conductors. In particular, for the preparation of samples suitable for *in situ* TEM investigation of SSEs, FIB-based preparation techniques are the standard approach, not only for micro-electro-mechanical system–based *in situ* setups [18] but also for scanning tunneling microscopy-based *in situ* TEM approaches [15]. While the impact of electron and ion beams on the structure and defects in semiconductor materials and devices has been extensively investigated, e.g. the influence of acceleration voltage, dose and dose rate, as well as various scanning strategies [19,20], the influence of FIB processing including SEM imaging on solid electrolytes is less well explored.

In addition to the well-known air and moisture sensitivity of many battery materials [21] and charging problems of SSEs due to their low electric conductivity [22,23], a fundamental issue is inherent damage by electron and ion beams on the structure, chemistry and oxidation state of battery materials [24]. Understanding and mitigating those challenges is essential for TEM analysis to investigate the real solid–solid interfaces in batteries. This becomes even more critical for *in situ* investigations, where it is often unknown how preparation-induced changes affect the electrochemical processes to be studied. For example, Lee et al. showed that metallic Li can be protected from morphological and significant chemical changes by performing the FIB sample processing under cryogenic conditions [25]. This enabled the investigation of the interfacial evolution between Li and a SSE during electrochemical cycling of a cross-section through a compressed ASSB.

Here, we report on the morphological, structural and chemical changes of three common SSEs, lithium phosphorus oxynitride (LiPON), Na-β"-alumina (BASE) and Na_3.4_Si_2.4_Zr_2_P_0.6_O_12_ (NaSICON), induced by SEM imaging and FIB processing and introduce an efficient cryogenic FIB approach to prevent significant changes during TEM sample preparation to enable reliable *in situ* and *ex situ* analysis of these materials in the TEM.

## Materials

Three common oxide-based SSEs have been investigated: pellets of commercial Na-beta”-alumina (BASE) (Ionotec Ltd., 14 Berkeley Court, Manor Park, Runcorn, Cheshire WA7 1TQ, UK) and NaSICON prepared according to Ma et al. [26] as examples for Na^+^ ion conductors as well as LiPON thin films (prepared by the group of Wolfram Jägermann at Technical University Darmstadt, Alarich-Weiss-Straße 2 64287 Darmstadt) [27] as an example for a Li^+^ ion conductors used in Li ASSBs.

## Methods

**Fig. 1.**
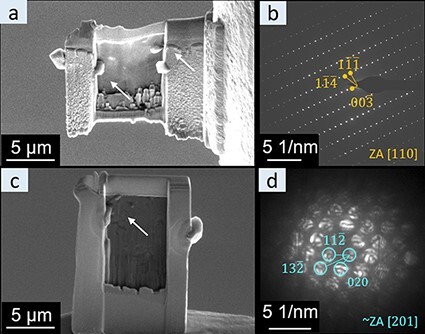
Working procedures of different FIB processes.

Standard and cryogenic FIB sample preparations have been performed using a Strata 400 S (FEI Company, Third Avenue 168, 02451, Waltham, MA, USA) DualBeam FIB and an Auriga 60 CrossBeam FIB (Zeiss, Carl-Zeiss-Strasse 22, 73447, Oberkochen, Germany). In addition to the cryogenic FIB process, a self-made stub with the specimen and TEM grid position was made for the liquid nitrogen (N_2_) cooling stage (Gatan Inc., 5794 W. Las Positas Blvd., Pleasanton, CA 94588, USA) as shown in Fig. 1. For BASE and NaSICON preparation, the samples were coated with a nominally 100-nm thick gold (Au) layer in a sputter coater (Quantum Design GmbH, Im Tiefen See 58, 64293, Darmstadt, Germany) on the SSE pellets and the silver paste was used to connect the surface of specimens and the SEM stubs to improve the electric conductivity for SEM/FIB imaging. Afterward, we performed both standard (here labeled s-FIB) and cryogenic FIB processing as shown in Fig. 1. The preparation labeled s-FIB follows standard FIB procedures [28] at room temperature (RT). Platium (Pt) was first deposited using the electron beam with an acceleration voltage of 5 kV and a current of 1.6 nA. The total electron dose for the deposition was around 8.1 × 10^8^ e/nm^2^. Afterward, Pt deposition was performed using FIB with a total dose of around 1.4 × 10^4^ Ga^+^/nm^2^. The trench milling and cutting of the lamella from the bulk were carried out with a total ion dose of 1.7 × 10^5^ Ga^+^/nm^2^ at 30 kV and a current of 9.3 nA to obtain a lamella with a thickness of around 2.5 μm. The lamella was transferred to the TEM grid with a micromanipulator. The lift-out and transfer process required around 1200 e/nm^2^ for SEM imaging and 400 Ga^+^/nm^2^ for attaching/removing the micromanipulator. For the final thinning and polishing, a dose of around 6.2 × 10^4^ Ga^+^/nm^2^ at 30 kV was used. At the end, to remove redeposited material and Ga ions from the surface of the TEM lamella, the specimen was briefly cleaned using a 5-kV ion beam. SEM imaging to support the milling and polishing procedures (including intermittent viewing and patterning) was conducted at 5 kV and with a current of 1.6 nA and a viewing frequency of 1 Hz. The final dose was estimated to be 1200 e/nm^2^ for the milling and polishing procedures. The detailed parameters are listed in Table 1.

**Table 1.** Work parameters for the different FIB processing conditions

		Working temperature/°C				
Step	FIB process	s-FIB	c-cryo-FIB	rt-cryo-FIB	Acceleration voltage and beam current	The angle between specimens' surface and the ion or electron beam/degree
1	Pt deposition	RT			Electron beam: 5 kV/1.6 nA	0
					Ion beam: 30 kV/280 pA	52 (Strata) 54 (Auriga)
2	Trench milling	RT	−184	RT	Ion beam: 30 kV/9.3 nA for s-FIB/rt-cryo-FIB and 16 nA for c-cryo-FIB	0
3	Lift-out and glue to TEM grid	RT			Electron beam: 5 kV/1.6 nA	0
					Ion beam: 30 kV/280 pA	52 (Strata) 54 (Auriga)
4	Thinning lamella	RT	−184	−184	Ion beam: 30 kV/93–430 pA for s-FIB and 240 pA for c-cryo-FIB and rt-cryo-FIB	Upside: −1.5 Downside: 1
5	Cleaning surface	RT	−184	−184	Ion beam: 5 kV/48 pA	Upside: −7 Downside: 1.5

The preparation of labeled c-cryo-FIB was performed analogously, but both the trench milling and the final polishing procedures were performed under cryogenic conditions (−184°C) using a liquid N_2_ cooling stage (Gatan Inc.). For the sample-labeled rt-cryo-FIB only, the final polishing was performed at −184°C, but the trench milling was performed at RT. In all cases, the doses and dose rates were similar. In all cases, the micromanipulator (OmniProbe 200 at Strata, OmniProbe 400 at Auriga) was kept at RT and the gas injection system was heated to 80°C. This allowed for cryogenic preparation of lift-out samples, using a regular FIB only equipped with a cryogenic stage.

Typically, the ion beam effects are considered to understand damage induced during FIB preparation. However, also, the electron beam in the SEM can lead to significant changes of materials [29,30]. To investigate electron beam effects in the SEM for SSEs in more detail, we performed a systematic series of dose experiments at various acceleration voltages (0.5–30 kV) using the SEM column inside the Strata 400 DualBeam FIB for LiPON thin films as well as the BASE pellet sample. For this analysis, we simply evaluated the morphological changes in the sample with increasing dose during continuous imaging at a fixed magnification.

A probe-corrected Themis 300 TEM (Thermo Fisher Scientific, 5350 NE Dawson Creek Dr, Hillsboro, OR 97124, USA) operated at 300 kV was used for TEM analysis of the (cryo) FIB-prepared samples. The electron beam diameter was nominally 170 pm with a convergence angle of 30 mrad and a screen current of 70 pA. High-angle annular dark-field scanning transmission electron microscopy (HAADF-STEM) imaging with energy-dispersive X-ray spectroscopy (EDS) (Super-X EDS detector) and electron diffraction was used to characterize the composition and microstructure of the TEM lamellas prepared by different FIB processes. The dose for one EDS map was around 2000 e/nm^2^. The Brown-Powell ionization cross-section model was used for the EDS quantification after second-order multi-polynomial modeling and subtraction of the background; the sample thickness and density were estimated for adsorption correction. Prior to TEM characterization, the TEM lamellas were cleaned by an argon–oxygen plasma using a 1070 Plasma Cleaner (Fischione Inc., 9003 Corporate Circle Export, PA 15632, USA) to remove the carbon compounds adsorbed on the surface. The FIB-processed SSE specimens were kept in a glove box for intermediate storage due to their sensitivity to humidity. Long-term exposure to air leads to a loss of sodium (Na) from the SSE [31]. However, the short transfer from the glove box to the TEM through air turned out not to be critical.

## Results and discussion

**Fig. 2.**
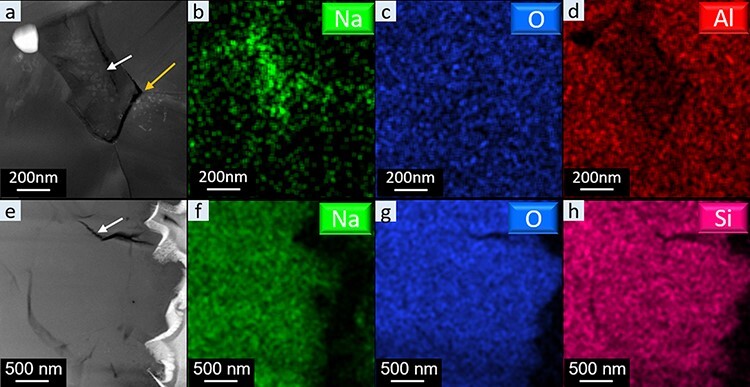
BASE and NaSICON fabricated by s-FIB; (a) SEM image of the BASE during the thinning procedure; (b) selected area electron diffraction of BASE; (c) SEM image of NaSICON during the thinning procedure; (d) nanobeam electron diffraction of NaSICON.

During standard (s-FIB) TEM preparation of both BASE and NaSICON, we noticed whiskers growing from the frame of the lamella and the thinned region as highlighted in Fig. 2a and c. The whiskers on the frame grow as the thinning of the central area progresses as shown in Supplementary Fig. S1. In the thinned region, this growth was not as obvious as further thinning partially removed the developing whiskers. However, whiskers tend to develop again at the same position after removing them by further thinning. Based on the fairly well-defined polyhedral shape, we assume that the whiskers where originally metallic Na, which oxidized during sample transfer into the TEM [32]. Despite the Na extraction from the SSE, the basic crystalline structure of the SSEs is well maintained as can be seen from the electron diffraction data (Fig. 2b and d), which is in excellent agreement with the NaSICON and BASE structure. We expect that the specific physical properties of the SSEs, in particular the high mobility of Na^+^ ions and the extremely low electronic conductivity, contribute to the development of Na whiskers during the ion-polishing procedure, while this effect is typically not observed in electrode materials. Radiolysis of ceramics typically leads to a displacement of atoms in the anion sublattice [33], which further leads to a loss of oxygen (in the case of oxide-based ceramics) [30] and ultimately to a damage of the crystalline lattice [33,34]. Nevertheless, even during our RT sample preparation, the damage to the crystal lattice was apparently not significant enough to see noticeable changes in the diffraction patterns. However, the Na located in between the cation sublattices is highly mobile in SSEs. In analogy to the electric field-induced damage mechanism suggested by Jiang et al. in STEM [22,23], we suggest that the electric field induces Na^+^ ion migration during FIB processing and SEM imaging as well. The Na^+^ ions in the bulk are driven to migrate either directly in the electric field gradient of the ion/electron beam as well as towards the sample surface due to the electric field developing in the insulating sample or by the accumulation of surface charges from the SEM or FIB imaging. Once at the surface, the Na ions can be reduced by surface electrons. This fits to the nucleation and growth of Na whiskers observed mostly at the edges of the thicker frame of the TEM lamella, where the strongest charging is expected and a rich Na reservoir is present in close vicinity. In contrast, during the trench milling procedure, we barely observed growth of Na whiskers on the surface of the specimen even though much larger ion doses have been employed. This is presumably related to the thick Au coating on the specimen surface, which is further connected via silver paste to the ground, thus providing good electrical contact and preventing significant charge accumulation at the surface both for the bulk specimen and the (still thick and well-connected) TEM lamella [35]. Therefore, the main driving force for Na ion migration and Na whisker growth is not present during this step.

To understand the influence of the standard FIB process on the SSEs, TEM characterization of the s-FIB-fabricated lamellas has been performed. Surprisingly, the selected area electron diffraction pattern of the BASE TEM lamella (Fig. 2b) can be clearly indexed as the [110] zone axis of Na-beta”-alumina (based on ICSD_200990) (Supplementary Table S1). This indicates that the typical layer structure of the BASE material is maintained after the s-FIB process. Moreover, the lattice parameters are not strongly affected by the Na loss, which is in agreement with observations during synthesis of BASE with various Na contents [36]. Similarly, the nanobeam electron diffraction of NaSICON (Fig. 2d) can be indexed well as the [$\bar 101$] zone axis of the monoclinic NaSICON structure [26] (based on ICSD_473). This indicates that the Na whisker growth during FIB thinning does not significantly influence the crystal structure of the SSEs and the Na ion transport path is presumably maintained after ion beam milling and polishing. A slight oxygen loss reported previously for various other oxides [29], and ceramics [30] can presumably be quickly recovered during the transfer of the TEM lamella from the FIB to the TEM [29], further helping to recover/maintain the Na ion transport paths.

**Fig. 3.**
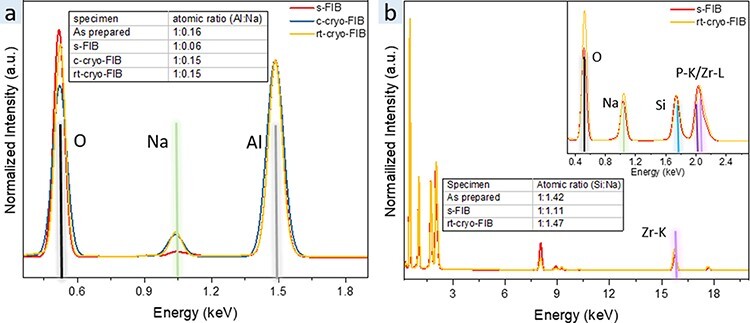
S-FIB-fabricated TEM lamellas of BASE and NaSICON; (a–d) HAADF-STEM image and EDS net intensity maps of Na, O and Al of BASE; (e–h) HAADF-STEM image and EDS maps of the integrated intensity for Na, O and Si of NaSICON.

At higher magnification, HAADF-STEM images and low-dose EDS elemental maps of BASE (Fig. 3a-d) show a reduced aluminum signal, indicating a thinner region compared to the adjacent grains, suggesting that this region was next to a void prior to specimen polishing, while small particles can be seen in the vicinity of voids and grain boundaries. Looking at the corresponding Na map, the small particles can be identified as Na (oxide), which originated from Na whiskers after the thinning procedure. This indicates that the Na whiskers preferentially grow at voids and grain boundaries during the thinning procedure. This has also been observed during *in situ* TEM studies looking at the influence of grain boundaries on Na ion migration [37]. Figure 3a also shows a triple boundary (right arrow), which presumably turned into a crack after thinning. This can be attributed to strain induced by the growth of Na whiskers at the triple boundary [37,38]. This suggests that the growth of the Na whiskers may also lead to some microstructural changes during thinning. Similar to the s-FIB-processed BASE material, Na was also found in the vicinity of voids/gaps of s-FIB-processed NaSICON TEM lamella as indicated by the white arrow in Fig. 3e and f. In addition, local Na fluctuations are also visible in Fig. 3e and f and Supplementary Fig. S2. In the vicinity of the Au coating layer, a significantly reduced Na content was observed in some NaSICON specimens due to FIB-induced alloying of Na and Au.

**Fig. 4.**
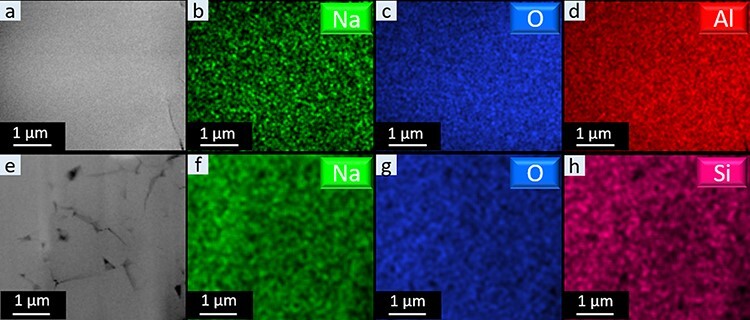
EDS spectra of different FIB-fabricated BASE (a) and NaSICON samples (b).

Although the crystal structure of the BASE and NaSICON material is maintained after the s-FIB TEM sample preparation, the Na whiskers growing from the frame of the lamella have a strong influence on the Na content of the thinned area due to the high mobility of Na^+^ ions in the SSEs. The Na content of the thinned area is critical for the application of the SSE due to the altered ionic conductivity, activation energy and driving force for ion migration. This, in turn, will affect *in situ* TEM experiments of oxide-SSE-based micro-/nanobatteries [36,39]. STEM-EDS analysis of the thinned area of the BASE and NaSICON lamella (Fig. 4a and b) reveals that the Na content of the thinned lamella is significantly reduced when looking at the Al:Na (BASE) and silicon:Na (NaSICON) ratio. The atomic ratio between Al and Na of the bulk BASE is 1:0.16, whereas in the s-FIB-fabricated BASE lamella, it is reduced to 1:0.06. The atomic ratio of Si and Na in bulk NaSICON is 1:1.42 [26], whereas in the s-FIB-fabricated NaSICON, it is reduced to 1:1.11. This indicates that around 60% of Na is lost in the thinned BASE lamella due to Na whiskers growth induced by the ion beam, and around 20% Na is lost in the thinned NaSICON lamella.

**Fig. 5.**
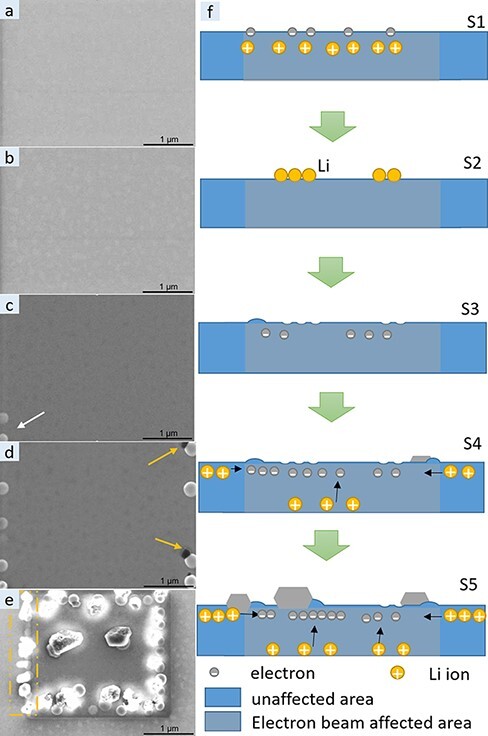
Rt-cryo-FIB-fabricated TEM lamellas of BASE and NaSICON; (a–d) HAADF-STEM image and low-dose EDS net intensity maps of Na, O and Al of BASE; (e–h) HAADF-STEM image and low-dose EDS net intensity maps of Na, O and Si of NaSICON.

As the crystal lattice of both BASE and NaSICON is maintained during FIB preparation and the most critical change during s-FIB fabrication is Na loss due to Na migration, cryogenic preparation should help to maintain the chemical composition by decreasing the Na^+^ ion mobility. Ideally, the whole preparation should be performed under cryogenic conditions, but with a standard FIB, this is not possible. Compared to the necessary ion dose for the trench milling and lamella thinning, the cut-off of the lamella during the lift-out process requires significantly lower dose. Therefore, the lift-out process is expected to have only a small influence on the overall lamella preparation. Hence, in this work, we used an FIB system equipped with a liquid N_2_ cooling stage to maintain cryogenic conditions for trench milling and thinning (c-cryo-FIB), whereas the Pt electron and ion deposition as well as the lift-out procedures were carried out at RT. Since we did not notice Na wire growth during trench milling, we further tried to simplify the fabrication procedures by applying cryogenic conditions only during TEM lamella thinning and polishing (rt-cryo-FIB). With this approach, we did not observe any Na whiskers forming during the cryogenic thinning procedure of both SSEs (Supplementary Fig. S3). A clean surface and uniform Na distribution were observed as shown in Fig. 5. Furthermore, the average Na content of the lamellas fabricated by c-cryo-FIB and rt-cryo-FIB has been determined by EDS (Fig. 4). With both cryogenic milling and polishing procedures, the Na to Al atomic ratio was improved to 1:6.5, which is close to the as-prepared bulk BASE composition, and after rt-cryo-FIB, the ratio was still around 1:6.6, close to the bulk composition. Similarly, in NaSICON, the Na to Si atomic ratio is at the same level as the as-prepared bulk NaSICON already when using cryogenic polishing. This indicates that the cryogenic conditions prevent a Na loss in the processed region. In addition, it suggests that controlling the cryogenic polishing during FIB processing is an efficient way to prepare TEM lamella of oxide SSEs.

Beside the ion beam-induced damage during FIB preparation, herein, we also investigated the material changes induced by the electron beam, which is necessary for viewing and patterning in SEM/FIB. It is widely accepted that charging effects occur in non-conductive materials, leading to a buildup of electric fields due to the accumulated surface electrons during SEM imaging [35,40]. Moreover, STEM imaging in the TEM is already known to facilitate Li^+^ ion diffusion and phase separation, which is induced by the gradient of the electric field in the material [41]. However, there is only limited information on the influence of the electron beam on the SSEs in the SEM. Therefore, we have explored morphological changes of SSEs in the SEM depending on dose and acceleration voltage of the electron beam.

**Fig. 6.**
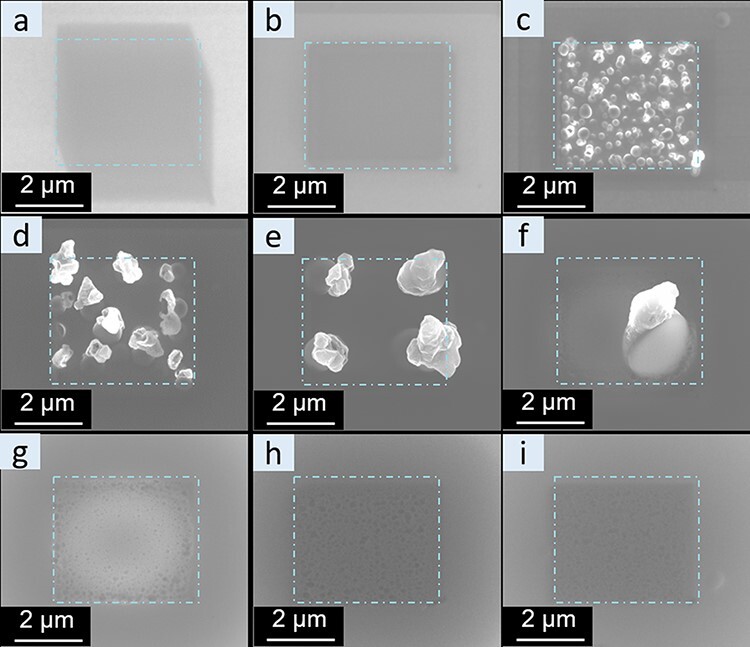
SEM imaging at 2 kV of a LiPON thin film with increasing dose and corresponding simplified model of the processes in the SEM: (a) initial thin film morphology; (b) after 22 e/nm^2^; (c) after 433 e/nm^2^ and (d) after 995 e/nm^2^; (e) demagnified SEM image after 3500 e/nm^2^; (f) simplified model of the structural changes observed with increasing dose in the SEM.

During SEM imaging of LiPON thin films, we noticed distinct morphological changes in the material with increasing dose. Starting at a dose as low as 22 e/nm^2^ at an acceleration voltage of 2 kV, first morphological changes appeared as small bright spots randomly distributed on the previously uniform thin film surface (Fig. 6). With increasing dose, these features disappeared gradually (video Supplementary information) and turned dark as shown in Fig. 6c. This morphological evolution is illustrated schematically in Fig. 6f. As a potential explanation for this behavior, we propose surface charging of the LiPON thin film due to the limited electrical conductivity inducing Li^+^ migration to the surface, where the Li^+^ ions are reduced to Li. The thin Li deposits might react with residual air/water in the FIB chamber and/or will be damaged by the continuous electron beam illumination, leading to the dark features observed during SEM imaging. In addition, the dose applied to the sample is at a level, where previous studies indicated first damage in the oxide structure to appear [29,30], which might also be responsible for the observed changes.

With further increasing dose, additional morphological changes become visible (Fig. 6c and d). First, larger spherical particles appear (white arrow in Fig. 6c), followed by whisker growth from the substrate at one edge of the particles (orange arrow in Fig. 6d). The whiskers are assumed to be metallic Li due to their well-defined polyhedral-like shape with characteristic faceting angles of around 120°. The spherical particles and the whiskers always start to grow at the left boundary of the scanning area followed by the right and top/bottom edge of the scanning, and only at much higher dose, they appear in the middle of the scanning area (see the Supplementary movie) at a dose of 2300 e/nm^2^. Surface charging leads to the largest electric field gradient at the boundary between the scanned and the surrounding area followed by preferential Li whisker growth there. The initial start of the growth on the left side of the scanned area (marked with the orange dashed rectangle in Fig. 6e) can be explained by the slightly higher dose applied there due to the fly-back time of the SEM beam during scanning. Both during the growth and at lower magnification (Fig. 6e), it becomes obvious that the whiskers grow pointing away from the edge of the scan frame, presumably due to the orientation of the electric field gradient. Only on the side marked in Fig. 6e, the whiskers grow to the left pointing away from the frame as the actual illuminated region starts there. Another contribution to the observed directed Li dendrite growth could also be the number of surface charges present, which would lead to faster Li reduction and higher growth speed in locations with a higher amount of surface charges.

**Fig. 7.**
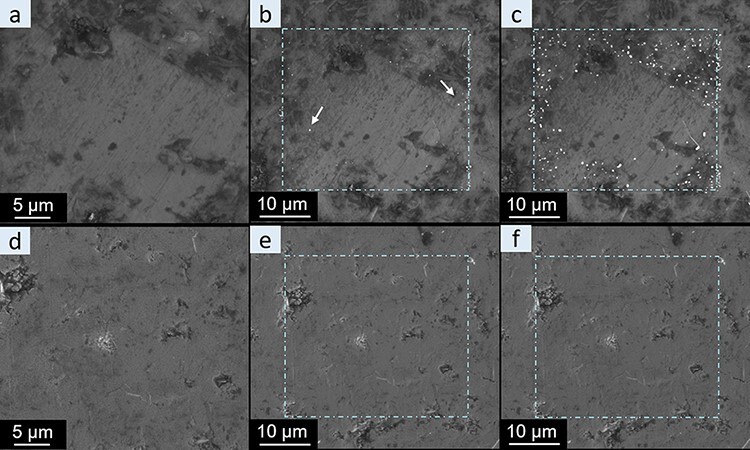
SEM imaging of a LiPON thin film at different acceleration voltages after applying a total dose of 3500 e/nm^2^ to the dash-dot rectangular region. At low voltages, some drift was unavoidable: (a) 0.5 kV, (b) 1 kV, (c) 2 kV, (d) 3 kV, (e) 5 kV, (f) 10 kV, (g) 15 kV, (h) 20 kV and (i) 30 kV.

In addition to the dependence on dosage, the influence of the acceleration voltage was also investigated as shown in Fig. 7. Applying the same final dose of 3500 e/nm^2^, SEM images of LiPON were acquired at different acceleration voltages. At low voltages of 0.5 and 1 kV, no whisker growth was observed, but only some surface changes built up, probably due to slight carbon contamination. At intermediate acceleration voltages of 2, 3, 5 and 10 kV, clear whisker growth was observed. The whisker density decreased and their size increased with increasing acceleration voltage in this range. This is presumably related to the distribution of surface electrons and the involved excitation volume. At low acceleration voltages (0.5 and 1 kV), surface charging leads to limited electric field buildup to induce Li^+^ ion migration. However, increasing acceleration voltages lead to an increase in the electron penetration depth and interaction volume with increased surface charging. Consequently, an increasing number of Li^+^ ions are affected to produce Li whiskers. The density changes are presumably a consequence of the difference in the nucleation and diffusion rates with increasing high tension. At higher acceleration voltages of 15, 20 and 30 kV, the morphological changes became less pronounced. A potential contribution could be the increasingly likely surface sputtering of Li at higher acceleration voltages, thus removing the Li growing on the surface. The threshold voltage for surface sputtering of Li has been estimated to be around 5–9 kV [42], fitting to the significantly reduced observation of Li wires above the acceleration voltage of 10 kV. Based on the morphological evolution at different acceleration voltages, extremely low acceleration voltages of 0.5 and 1 kV are the best conditions for SEM imaging of LiPON during FIB preparation to reduce electron beam–induced damage.

**Fig. 8.**
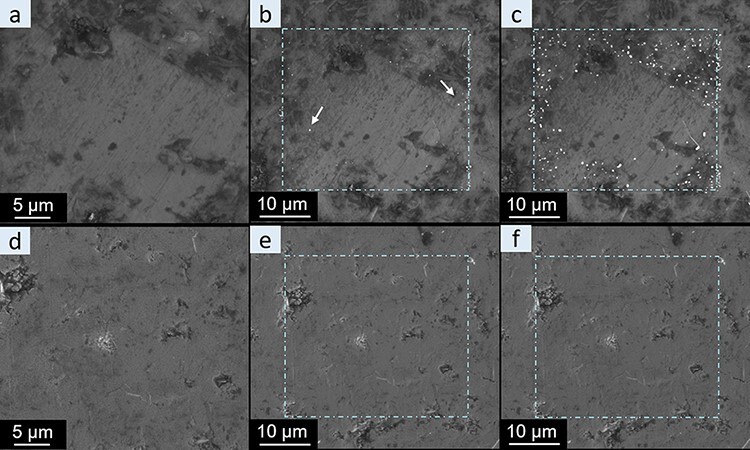
SEM images acquired at an acceleration voltage of 5 kV of pristine (a–c) and Au-coated (d–f) BASE pellets after exposure of the dash-dot rectangular regions to different electron dose: (a) pristine sample surface; (b) first Na whisker growth (indicated by the white arrows) at a total dose of ∼600 e/nm^2^; (c) significantly increased growth of Na whiskers at the edge of the scanned region at a total dose of ∼1200 e/nm^2^; (d). pristine Au-coated BASE pellet; (e) no whisker growth at a total dose of ∼600 e/nm^2^ and (f) ∼1200 e/nm^2^.

As an alternative approach, a metal coating is a well-known method to minimize the charging effect in the SEM [35]. Therefore, in this work, the BASE and NaSICON pellets were coated with Au for the TEM sample preparation. Here, we compared the behavior of Au-coated and Au-uncoated BASE pellets using the same dose conditions as earlier (Fig. 8). Without an Au coating, whiskers started to appear at a dose of around 600 e/nm^2^ (white arrows in Fig. 8b) and more whiskers grew around the edge of the scan area after a dose of around 1200 e/nm^2^ (Fig. 8c). However, the Au-coated sample did not show any whisker growth under the same dose condition (Fig. 8d–f), and even at a much higher dose of around 60 000 e/nm^2^, no whisker growth was observed (Supplementary Fig. S4). The suppression of whisker growth during SEM imaging of the Au-coated specimen is another indication that the whisker growth is related to surface charges and the resulting electric field. In turn, the Au coating is an efficient protection preventing metal ion migration in the SEM.

## Conclusion

We explored the influence of FIB processing and SEM imaging on oxide SSEs during imaging and TEM sample preparation. Li/Na whiskers grew from the SSE surface, induced by both the electron and the ion beam. We proposed that the local electric fields generated by the electron/ion beam and, more dominant, the charging of the insulating SSE samples is the driving force for Li/Na migration. When good electrical contact of the SSE is maintained, no significant Na loss was observed, but the final thinning and polishing procedures were critical. With typical RT FIB preparation, this resulted in a Na loss of around 60% in the case of BASE and 20% in the case of NaSICON. Nevertheless, the crystal structure of both SSEs was maintained during FIB preparation without noticeable changes in lattice parameters. To reduce Na migration during TEM sample preparation, cryogenic preparation with liquid N_2_ cooling was successful, resulting in TEM samples with an essentially maintained nominal composition. Even if only the final thinning and polishing were performed with liquid N_2_ cooling, the preparation of TEM samples without noticeable changes was successful. This is a very efficient way to prepare sensitive samples.

Not only the Ga^+^ ion beam is changing the sample, but also SEM imaging can induce significant Li^+^/Na^+^ ion migration in SSEs. Whisker growth was observed during SEM imaging for both LiPON thin films and BASE ceramics. The damage is dependent on acceleration voltage and results in significant whisker growth between acceleration voltages of 2 and 10 kV. The observed morphological changes are also in agreement with surface charging as the main driving force for ion migration. Applying a protective layer of Au to strongly reduce surface charging thus efficiently reduces the damage during SEM imaging and thus helps during SEM characterization as well as for a good TEM sample preparation by FIB.

## Supplementary Material

dfac064_Supp

## Data Statement

The original data are available at KITOpen through the DOI: 10.5445/IR/1 000 152 787.
