# Supplementary material for: Exploring the influence of focused ion beam processing and scanning electron microscopy imaging on solid-state electrolytes
Source: Microscopy (Oxf). 2022 Nov 21;72(4):326–35. doi: 10.1093/jmicro/dfac064 (PMC10402911; doi:10.1093/jmicro/dfac064)
Supplement: dfac064_Supp [file dfac064_supp.zip › suppl_data/FIB Battery - SI.docx]

**Exploring the influence of FIB processing and SEM imaging on solid-state electrolytes**

**Supplementary information**

Ziming Ding, Yushu Tang, Venkata Sai Kiran Chakravadhanula, Qianli Ma, Frank Tietz, Till Ortmann, Yuting Dai, Torsten Scherer, Christian Kübel*

Ziming Ding, Institute of Nanotechnology (INT), Karlsruhe Institute of Technology (KIT), 76344 Eggenstein-Leopoldshafen, Germany; Technische Universität Darmstadt, 64289 Darmstadt, Germany. +4972160834346 [ziming.ding@kit.edu](mailto:ziming.ding@kit.edu)

Yushu Tang, Institute of Nanotechnology (INT), Karlsruhe Institute of Technology (KIT), 76344 Eggenstein-Leopoldshafen, Germany; +4972160828932 [yushu.tang@kit.edu](mailto:yushu.tang@kit.edu)

Venkata Sai Kiran Chakravadhanula, Skyroot Aerospace, Hyderabad, Telangana, India; [cvskiran@gmail.com](mailto:cvskiran@gmail.com)

Qianli Ma, Forschungszentrum Jülich GmbH, Institute of Energy and Climate Research, Materials Synthesis and Processing (IEK-1), 52425 Jülich, Germany; +492461/61-9705 [q.ma@fz-juelich.de](mailto:q.ma@fz-juelich.de)

Frank Tietz, Forschungszentrum Jülich GmbH, Institute of Energy and Climate Research, Materials Synthesis and Processing (IEK-1), 52425 Jülich, Germany; +492461/61-5007 [f.tietz@fz-juelich.de](mailto:f.tietz@fz-juelich.de)

Till Ortmann, Institute for Physical Chemistry, Justus Liebig University Giessen, 35392 Giessen, Germany; +4964199-34521 [Till.Ortmann@phys.chemie.uni-giessen.de](mailto:Till.Ortmann@phys.chemie.uni-giessen.de)

Yuting Dai, Institute of Nanotechnology (INT) and Helmholtz Institut Ulm (HIU), Karlsruhe Institute of Technology (KIT), 76344 Eggenstein-Leopoldshafen, Germany; Technische Universität Darmstadt, 64289 Darmstadt; +4972160828932 [yuting.dai@partner.kit.edu](mailto:yuting.dai@partner.kit.edu)

Torsten Scherer, Institute of Nanotechnology (INT), Karlsruhe Institute of Technology (KIT), 76344 Eggenstein-Leopoldshafen, Germany; +4972160828365 [torsten.scherer@kit.edu](mailto:torsten.scherer@kit.edu)

Christian Kübel*, Institute of Nanotechnology (INT), Helmholtz Institute Ulm (HIU), and Karlsruhe Nano Micro Facility (KNMF), Karlsruhe Institute of Technology (KIT), 76344 Eggenstein-Leopoldshafen, Germany; Technische Universität Darmstadt, 64289 Darmstadt; +4972160828970 [christian.kuebel@kit.edu](mailto:christian.kuebel@kit.edu)


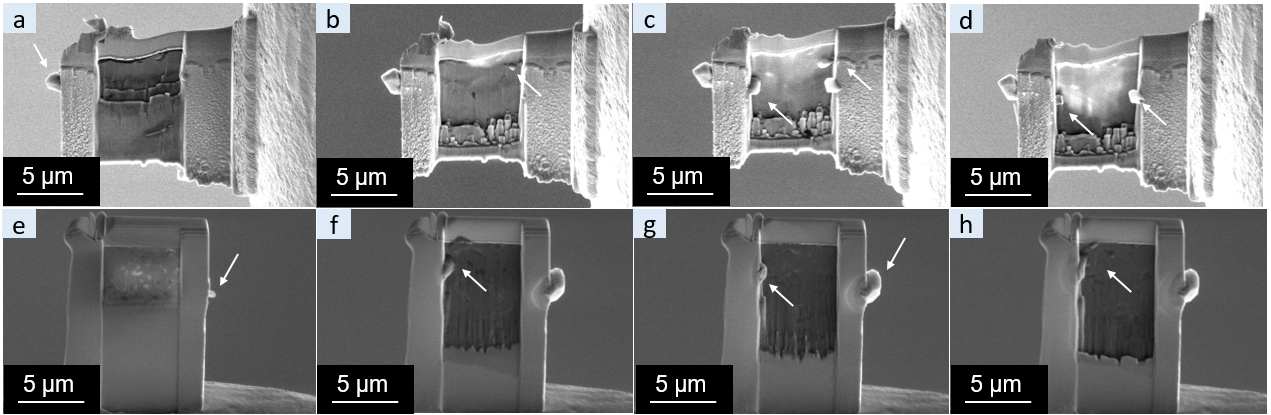


**Figure S1**. TEM lamellas fabricated by s-FIB; a-d. Na-beta’’-alumina (BASE); e-h. Na_3.4_Zr_2_Si­­_2.4_P_0.6_O_12_ (NASICON).


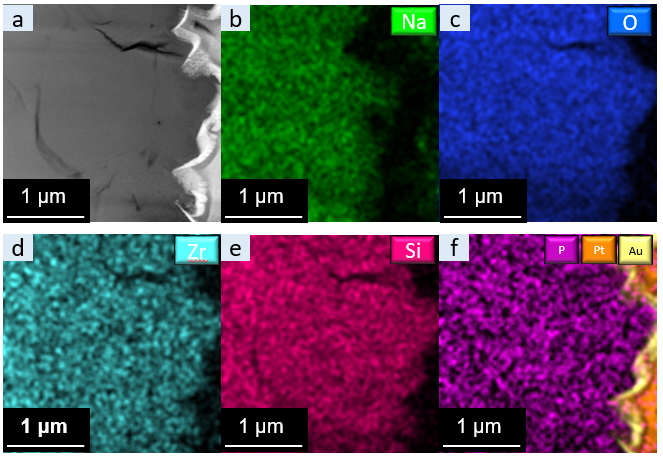


**Figure S2.** HAADF-STEM EDS elemental net intensity mapping of s-FIB fabricated NaSICON lamella.

**Table S1** Lattice spacing for BASE and NASICON. The references for BASE and monoclinic NASICON are based on ICSD_200990 and ICSD_473, respectively. The slight variations can be attributed to slight calibration and measurement errors.

|  | Lattice plane | Reference lattice spacings/nm | ED based lattice spacings/nm |
| --- | --- | --- | --- |
| BASE | ($00\bar{3}$) | 1.12 | 1.12 |
|  | ($1\bar{1}\bar{1}$) | 0.48 | 0.47 |
|  | ($1\bar{1}\bar{4}$) | 0.42 | 0.41 |
|  | ($1\bar{1}2$) | 0.47 | 0.46 |
|  | ($\bar{1}1\bar{5}$) | 0.39 | 0.39 |
| NaSICON  (monoclinic phase) | ($11\bar{2}$) | 0.40 | 0.41 |
|  | ($13\bar{2}$) | 0.25 | 0.26 |
|  | ($020$) | 0.45 | 0.47 |
|  | ($0\bar{2}0$) | 0.40 | 0.43 |
|  | ($1\bar{3}\bar{2}$) | 0.25 | 0.26 |


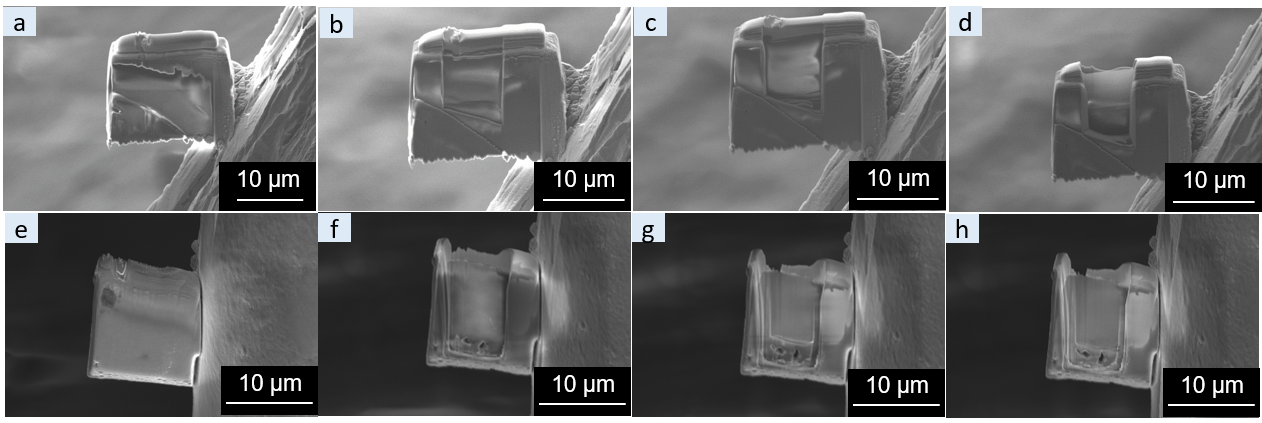


**Figure S3**. TEM lamella fabricated by cryogenic FIB; a-d. BASE; e-h. NaSICON.


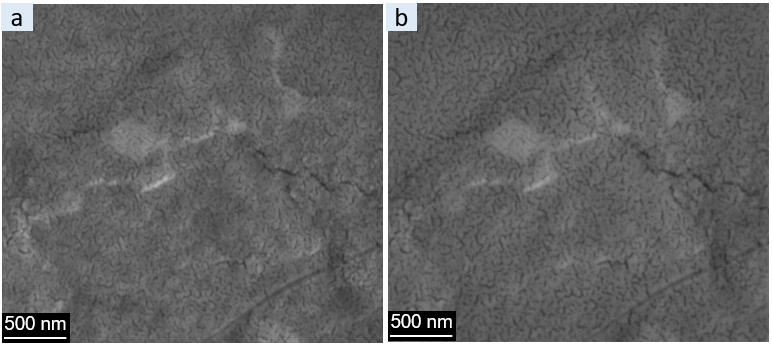


**Figure S4.** SEM imaging at an acceleration voltage of 5 kV for Au-coated BASE; a. initial state; b. after the total dose ~60000 e/nm^2^.
