# Supplementary material for: Exploring the influence of focused ion beam processing and scanning electron microscopy imaging on solid-state electrolytes
Source: Microscopy (Oxf). 2022 Nov 21;72(4):326–35. doi: 10.1093/jmicro/dfac064 (PMC10402911; doi:10.1093/jmicro/dfac064)
Supplement: dfac064_Supp [file dfac064_supp.zip › suppl_data/Table S1.docx]

**Table S1** Lattice spacing for BASE and NASICON. The references for BASE and monoclinic NASICON are based on ICSD_200990 and ICSD_473, respectively. The slight variations can be attributed to slight calibration and measurement errors.

|  | Lattice plane | Reference lattice spacings/nm | ED based lattice spacings/nm |
| --- | --- | --- | --- |
| BASE | ($00\bar{3}$) | 1.12 | 1.12 |
|  | ($1\bar{1}\bar{1}$) | 0.48 | 0.47 |
|  | ($1\bar{1}\bar{4}$) | 0.42 | 0.41 |
|  | ($1\bar{1}2$) | 0.47 | 0.46 |
|  | ($\bar{1}1\bar{5}$) | 0.39 | 0.39 |
| NaSICON  (monoclinic phase) | ($11\bar{2}$) | 0.40 | 0.41 |
|  | ($13\bar{2}$) | 0.25 | 0.26 |
|  | ($020$) | 0.45 | 0.47 |
|  | ($0\bar{2}0$) | 0.40 | 0.43 |
|  | ($1\bar{3}\bar{2}$) | 0.25 | 0.26 |
