# Supplementary figures and images for: Exploring the influence of focused ion beam processing and scanning electron microscopy imaging on solid-state electrolytes
Source: Microscopy (Oxf). 2022 Nov 21;72(4):326–35. doi: 10.1093/jmicro/dfac064 (PMC10402911; doi:10.1093/jmicro/dfac064)

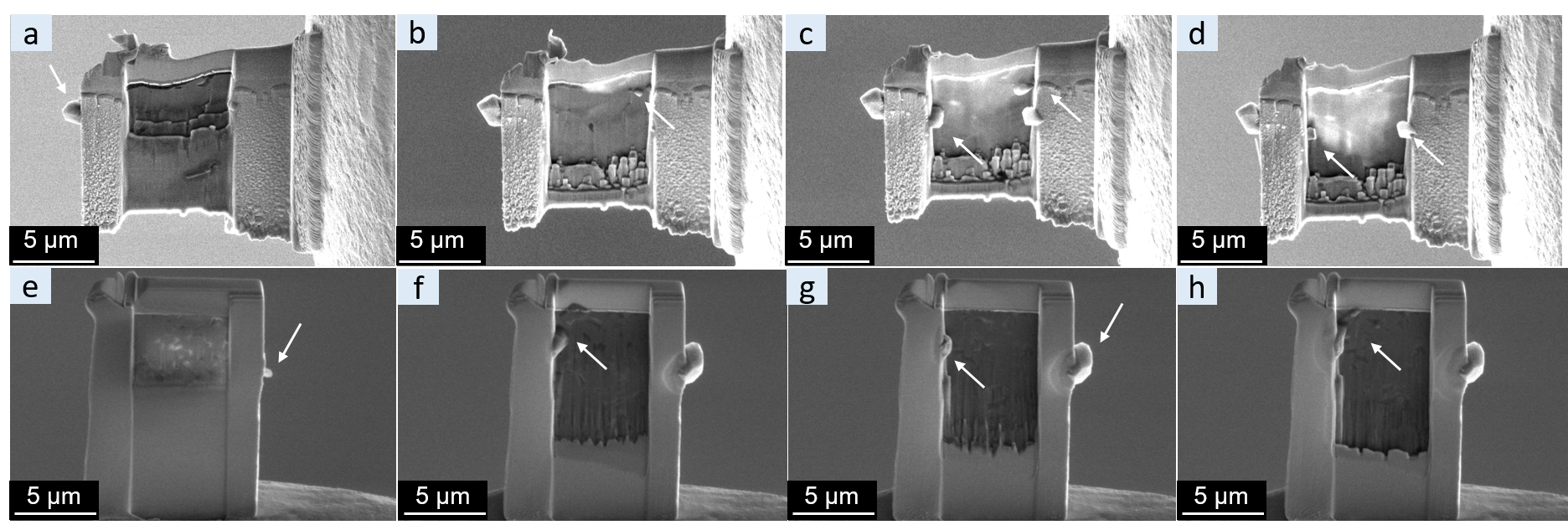

Supplement: dfac064_Supp [file dfac064_supp.zip › suppl_data/Figure S1.tif]

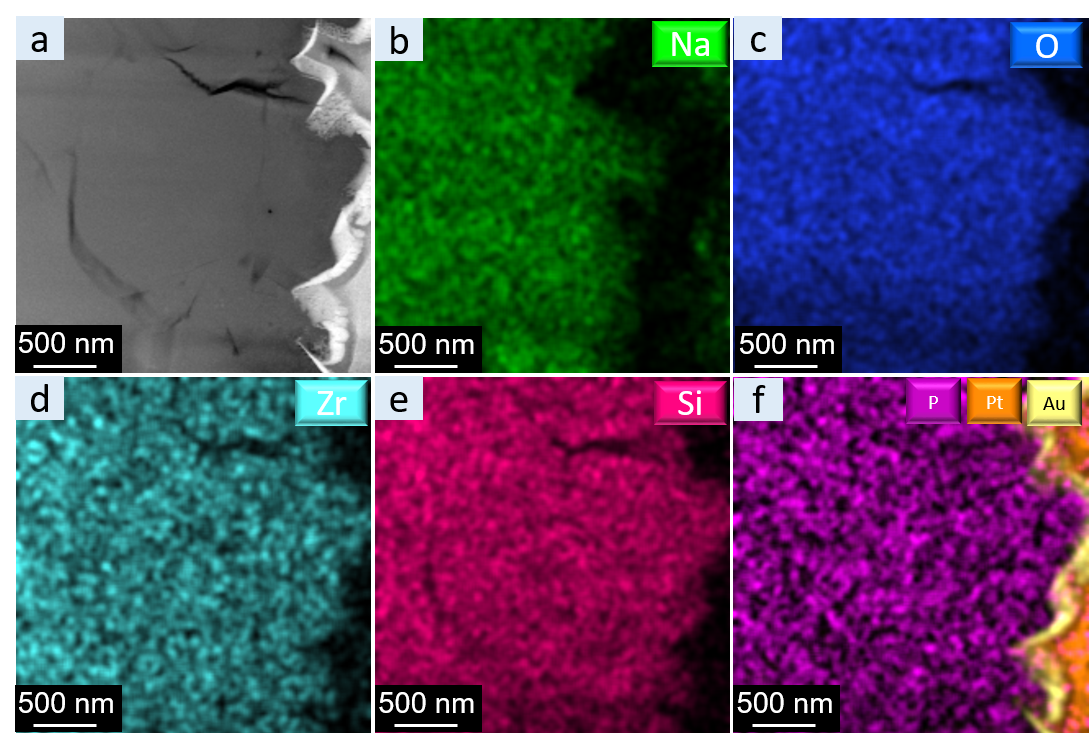

Supplement: dfac064_Supp [file dfac064_supp.zip › suppl_data/Figure S2.tif]

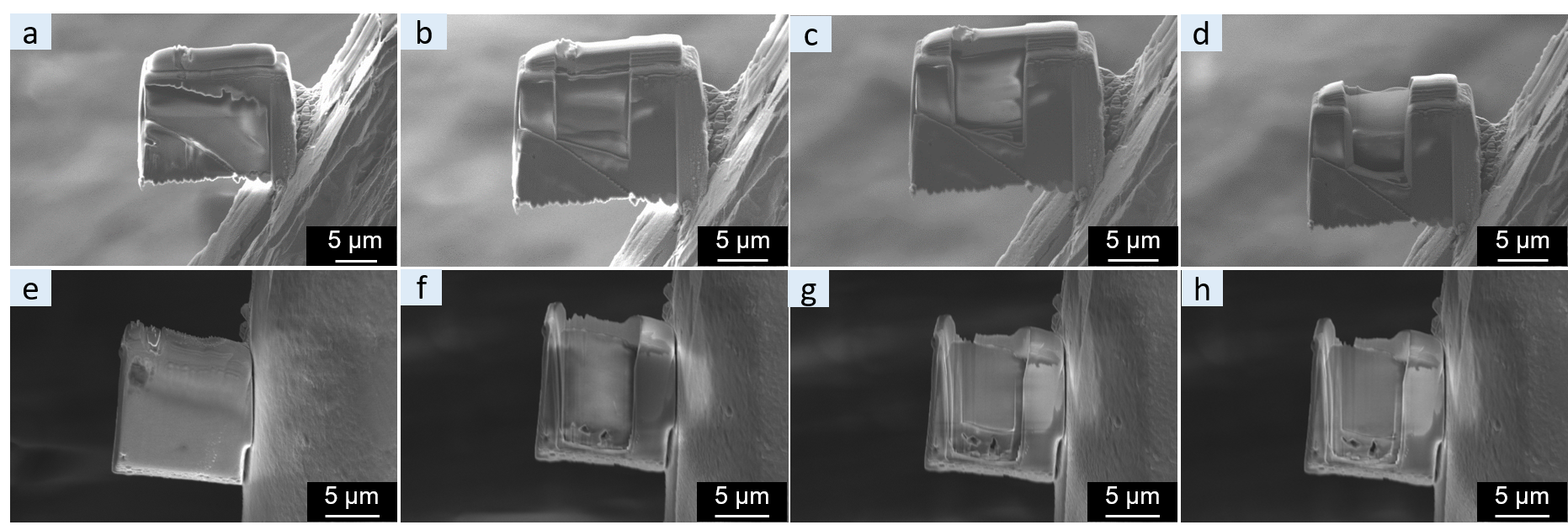

Supplement: dfac064_Supp [file dfac064_supp.zip › suppl_data/Figure S3.tif]

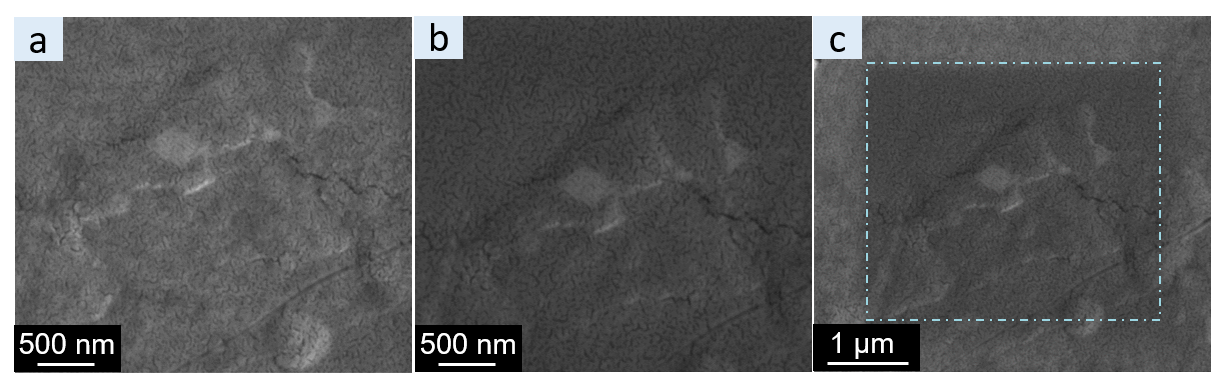

Supplement: dfac064_Supp [file dfac064_supp.zip › suppl_data/Figure S4.tif]
